# FishMitoCleaner: Automated Stop Codon Processing and Quality Control of Fish Mitochondrial Protein‐Coding Sequences

**DOI:** 10.1002/ece3.74557

**Published:** 2026-10-18

**Authors:** Shaoyu Yang, Peng Xu, Jingzhen Wang, Xiaohui Cai

**Affiliations:** ^1^ Guangxi Key Laboratory of Marine Environmental Disaster Processes and Ecological Protection Technology Beibu Gulf University Qinzhou China

**Keywords:** codon usage, fish genomics, mitochondrial genome, sequence preprocessing, stop codon

## Abstract

Vertebrate mitochondrial protein‐coding genes follow a different genetic code, in which TGA encodes tryptophan and a large number of genes terminate with incomplete stop codons completed post‐transcriptionally via polyadenylation. These features are often mishandled when coding sequences (CDS) downloaded from public databases are processed with standard tools, resulting in errors in downstream codon usage and evolution. Here we present FishMitoCleaner, a Python command line tool automating quality control and normalisation of fish mitochondrial CDS sequences in five steps: character validation, internal stop codon scanning, terminal TGA detection, stop codon processing and length normalisation. FishMitoCleaner cleaned 97.6% of input sequences in 22 fish species representing 7 orders and all 13 mitochondrial protein coding genes. Statistical comparison of RSCU values confirmed no bias in codon usage analysis (Welch's *t*‐test, all *p* > 0.05). FishMitoCleaner is a Python command that allows researchers in aquatic biology and fisheries science to perform rigorous and reproducible CDS quality control in a single command, and clean sequences ready to be analysed in downstream analyses. FishMitoCleaner is also applicable to mitochondrial CDS data from other vertebrate taxa.

## Graphical abstract

FishMitoCleaner automates quality control of fish mitochondrial protein‐coding sequences, correctly handling both complete and incomplete stop codons while protecting terminal TGA codons as tryptophan residues. This tool requires no programming expertise and produces analysis‐ready sequences in a single command, resolving common errors that bias downstream molecular evolution analyses.

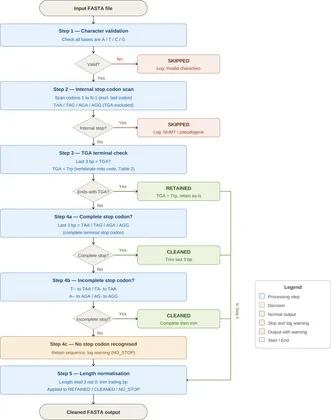

## 1 Introduction

The mitochondrial genome is one of the most widely used genetic markers in vertebrate biology. Fish mitogenomes are closed circular double‐stranded DNA molecules of about 16 kb size with 13 protein‐coding genes (PCGs), 22 transfer RNA genes (tRNAs), 2 ribosomal genes (rRNAs) and a D‐loop (Boore 1999). Due to their maternal inheritance, high copy number, fast evolution rate and lack of recombination, fish mitogenomes have been used to reconstruct phylogenetic information, species identification, population genetics and selection pressure estimation in fish and other vertebrates (Sun et al. 2024; Muhala et al. 2024; Zhang et al. 2024). With high‐throughput sequencing technology, fish mitogenomes have been published in public databases such as NCBI GenBank (Alvarenga et al. 2024; Veríssimo et al. 2025) and MitoFish (Zhu et al. 2023).

Quality control and normalisation of protein‐coding sequences (CDS) downloaded from public databases must be performed before downstream analyses can be performed. The vertebrate mitochondria use a different genetic code (NCBI Translation Table 2; https://www.ncbi.nlm.nih.gov/Taxonomy/Utils/wprintgc.cgi#SG2), which differs from the standard genetic code in several biologically important ways. These are often overlooked errors in CDS preprocessing (Osawa et al. 1989). First, TGA encodes tryptophan (Trp) in vertebrate mitochondria, rather than a stop signal; processing mitochondrial CDS with standard code tools will thus strip TGA‐terminal sequences and lose the terminal amino acid residue. Second, the recognised stop codons in vertebrate mitochondrial annotations include TAA, TAG and—in certain PCGs—AGA and AGG (Osawa et al. 1989). Third, and most complex in practice, most vertebrate mitochondrial PCGs end with incomplete stop codons: the 3′ end encodes only one T (T‐‐) or dinucleotide TA (TA‐) which is completed to a functional TAA stop codon after transcribing via polyadenylation (Ojala et al. 1981). This is a problem prevalent in fish; it has been observed in various teleost orders, including Siluriformes, Perciformes and Cypriniformes (Sun et al. 2024; Zhang et al. 2024; Zhang, Zhu, and Luo 2023).

These biological features directly contribute to systemic inconsistencies in CDS annotations in public databases. Bernt and colleagues showed that incomplete stop codons (T‐‐ or TA‐) are annotated inconsistently in RefSeq (some records contain the polyadenylation complete stop codon while others have only partial stop codon), and in some cases, a complete codon is annotated as a stop codon even though it is not a stop codon according to the designated translation table (Donath et al. 2019). Other than the inconsistency of annotations, downloaded CDS sequences may also contain in‐frame internal stop codons (a feature of nuclear copies of mitochondrial DNA [NUMT] insertions or sequencing and annotation errors; Donath et al. 2019) or IUPAC ambiguity codes (e.g., N, R, Y) that render sequences unsuitable for codon analyses. Furthermore, output sequences must have lengths that are strict multiples of three before Codon analysis (Ka/Ks calculation) or relative synonymous codon usage (RSCU) profiling can be performed. Failure to enforce this requirement introduces systematic errors.

There are several tools for mitochondrial genomics work such as MitoAnnotator for fish mitogenome annotation (Iwasaki et al. 2013), MITOS for metazoan mitogenome annotation (Donath et al. 2019) and MitoGeneExtractor for gene extraction from NGS libraries (Brasseur et al. 2023), but they are intended for genome annotation or sequence extraction. To our knowledge, no standalone tool exists for batch quality control and normalisation of already annotated vertebrate mitochondrial CDS sequences. In practice, researchers often have to use manual inspection or ad hoc scripts to deal with these issues for each data set, which is time consuming, inconsistent and difficult to reproduce when handling hundreds or thousands of sequences in large‐scale analysis.

To address this need, we present FishMitoCleaner, a Python‐based open source command line tool for the automatic preprocessing and quality control of fish and vertebrate mitochondrial CDS sequences. FishMitoCleaner follows the vertebrate mitochondrial genetic code and implements a five‐steps sequential pipeline covering character validation, internal stop codon scanning, terminal TGA protection, stop codon processing (complete and incomplete) and length normalisation. FishMitoCleaner requires no programming skills and produces clean, analysis ready sequences along with a processing report and a statistics figure in one command.

## 2 Methods

### 2.1 Tool Implementation

FishMitoCleaner is a single‐file Python command line tool (version 1.0.0) for Python 3.8 and above running on Windows, MacOS and Linux. FishMitoCleaner accepts multi‐sequence FASTA file input and processes each sequence individually in five steps (Figure 1). Third‐party dependencies include Biopython (≥ 1.70) for FASTA parsing and sequence handling, Matplotlib (≥ 2.2) figure generation and Tabulate package for formatted console output. All dependencies are checked at runtime; informative errors are shown if any package is missing or outdated.

**FIGURE 1:**
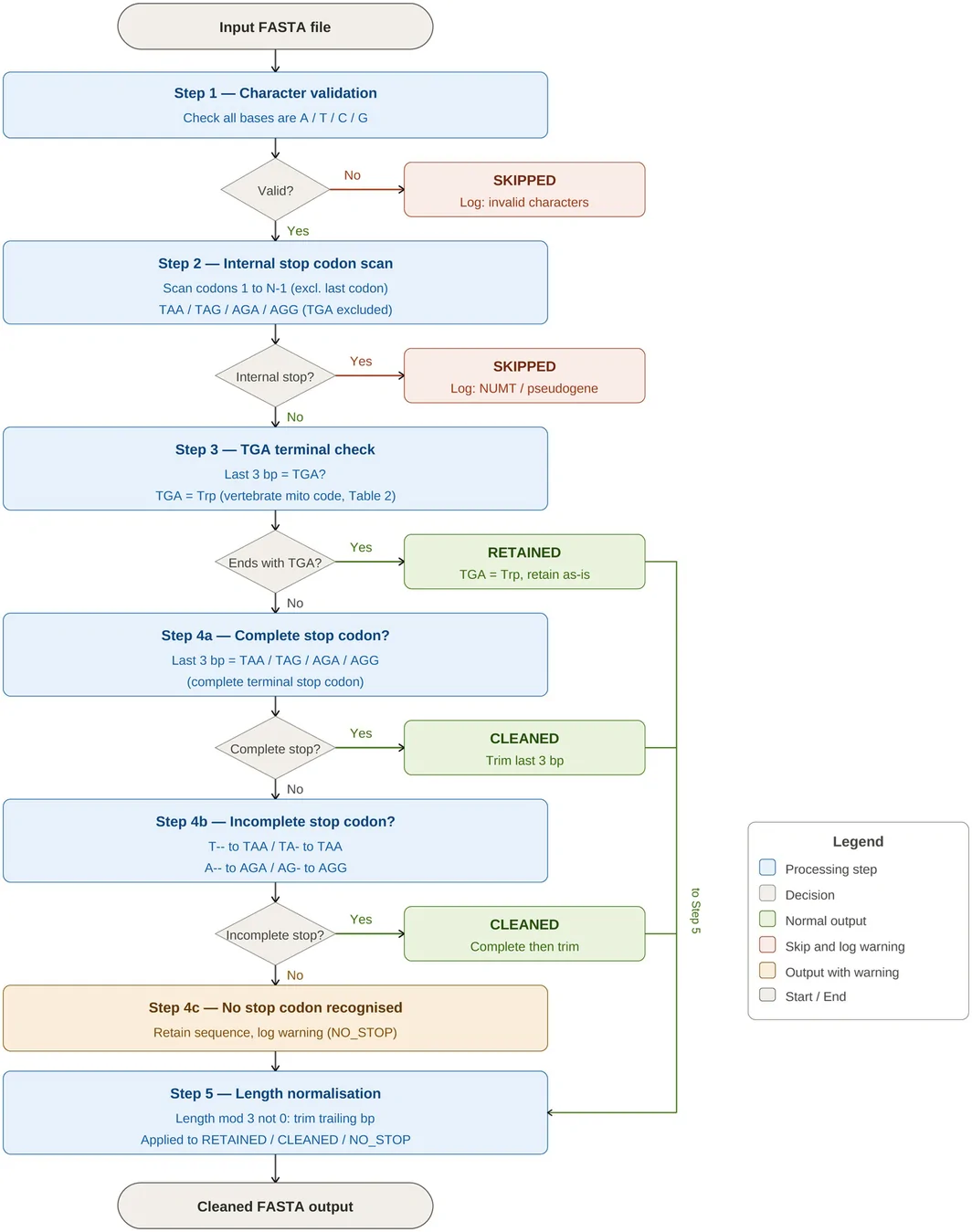
Overview of the FishMitoCleaner processing pipeline. The five‐step sequential workflow covers character validation, internal stop codon scanning, terminal TGA detection, stop codon processing (Steps 4a–4c) and length normalisation (Step 5). Blue boxes indicate processing steps; grey diamonds indicate decision points; green boxes indicate normal outputs; red boxes indicate sequences excluded with a warning; orange boxes indicate outputs retained with a warning. Each sequence is processed until it meets the criteria of a given step, at which point processing terminates.

### 2.2 Sequence Processing Pipeline

Each input sequence is converted to uppercase and processed through the following five sequential steps. Processing terminates and returns a result as soon as a sequence meets the criteria of any step.

#### 2.2.1 Step 1: Character Validation

The sequence is checked for non‐canonical bases using a regular expression with A, T, C, G. Sequences with IUPAC ambiguity codes (e.g., N, R, Y) or other non‐standard characters are marked as SKIPPED with the offending characters logged in the log, and excluded from all downstream analyses.

#### 2.2.2 Step 2: Internal Stop Codon Scan

Frames from 1 to N‐1 (all except the terminator) are scanned for vertebrate mitochondrial stop codons TAA, TAG, AGA, AGG. TGA is not considered because it encodes tryptophan under the vertebrate mitochondrial genetic code (NCBI Translation Table 2) (Barrell et al. 1979). Sequences with any in‐frame internal stop codon are marked as SKIPPED, since such sequences likely represent nuclear mitochondrial DNA segments or pseudogenes.

#### 2.2.3 Step 3: Terminal TGA Detection

The terminal three nucleotides (last3) are examined. If last3 equals TGA, the sequence is retained without trimming (status: RETAINED), because TGA encodes tryptophan in the vertebrate mitochondrial genetic code (Ojala et al. 1981). The sequence then proceeds to Step 5.

#### 2.2.4 Step 4a: Complete Stop Codon Removal

If last3 is one of TAA, TAG, AGA or AGG, the terminal three nucleotides are removed (status: CLEANED). The sequence proceeds to Step 5.

#### 2.2.5 Step 4b: Incomplete Stop Codon Repair

Vertebrate mitochondrial genes often terminate with a partial stop codon (T‐‐, TA‐, A‐‐ or AG‐) that is completed post‐transcriptionally by polyadenylation (Ojala et al. 1981; Donath et al. 2019). FishMitoCleaner recognises all four patterns via regular expression matching and removes the corresponding partial terminal bases (status: CLEANED). The sequence proceeds to Step 5.

#### 2.2.6 Step 4c: No Stop Codon Recognised

If the terminal sequence matches none of the above patterns, the sequence is retained with a warning (status: NO_STOP) and proceeds to Step 5.

#### 2.2.7 Step 5: Length Normalisation

All sequences reaching this step (RETAINED, CLEANED or NO_STOP) are trimmed if necessary to ensure their length is a strict multiple of three, as required for codon‐based downstream analyses (e.g., Ka/Ks calculation, RSCU analysis).

### 2.3 Installation and Usage

FishMitoCleaner requires Python 3.8 or higher and can be installed by downloading the source package from Zenodo and installing the dependencies with:

pip install biopython matplotlib tabulate

The tool is invoked from the command line. Three usage modes are supported:

1. Demo mode—run without arguments to automatically generate and process a built‐in demonstration dataset covering all eight sequence types:

python FishMitoCleaner.py

2 Standard mode—supply a FASTA file containing one or more CDS sequences to be cleaned:

python FishMitoCleaner.py ‐i input_sequences.fasta

3 Standard mode with custom output directory:

python FishMitoCleaner.py ‐i input_sequences.fasta ‐o output_directory/

The input FASTA file may contain any number of sequences. Each sequence is processed independently; the order of sequences in the file does not affect results. No pre‐sorting or pre‐alignment of sequences is required.

### 2.4 Output Files

Once done, FishMitoCleaner writes all results to the output directory (default FishMitoCleaner_output/) and prints a report per sequence to terminal. The outputs are summarised in Table 1. Each sequence is marked with one of five final status codes: CLEANED (stop codon cut), RETAINED (TGA terminal kept as‐is), NO_STOP (no recognised stop codon kept with warning), SKIPPED (invalid characters or internal stop codon detected), MOD3_FAIL (rare edge case where length normalisation cannot be completed).

**TABLE 1:** Output files generated by FishMitoCleaner v1.0.0.

Output file	Format	Resolution/type	Description
cleaned_sequences.fasta	FASTA	—	Cleaned sequences. Contains all sequences with status CLEANED, RETAINED or NO_STOP.
stats_figure.png	PNG	600 DPI	Summary statistics bar chart and pie chart showing counts and proportions of all processing categories.
stats_figure.pdf	PDF	Vector	Vector version of the statistics figure, suitable for journal submission.
Console report	Text	—	Per‐sequence processing table printed to the terminal, recording sequence ID, original length, terminal status, action taken, final status and any warnings.

### 2.5 Automated Testing

A test suite of 32 unit and integration tests was built using Python's built‐in unittest framework covering all processing steps and edge cases: valid and invalid character inputs, all four complete stop codon types, all four incomplete stop codon patterns, TGA terminal retention, internal stop codon detection and length normalisation. All 32 tests pass under Python versions 3.8 to 3.12. The test suite can be run with:

python tests/test_FishMitoCleaner.py

### 2.6 Validation Dataset

To evaluate FishMitoCleaner in real‐world scenarios, we downloaded complete mitochondrial genome sequences for 22 fish species from NCBI GenBank. These species represent seven orders: Pleuronectiformes, Salmoniformes, Cypriniformes, Perciformes, Anguilliformes, Siluriformes and Tetraodontiformes. The 13 PCG CDS sequences were extracted from each genome record based on annotated CDS features. A total of 286 sequences (22 species × 13 PCGs) were processed with FishMitoCleaner v1.0.0 using default parameters. Species names, taxonomic orders and GenBank accession numbers are in Table S1.

## 3 Results

### 3.1 Tool Workflow

FishMitoCleaner implements a five‐step sequential decision pipeline for vertebrate mitochondrial CDS sequences (Figure 1). Each input sequence is evaluated in order: (1) character validation, (2) internal stop codon scanning, (3) terminal TGA detection, (4a/4b/4c) stop codon classification and processing and (5) length normalisation. The pipeline handles all vertebrate mitochondrial DNA sequences, including TGA being recognised as tryptophan rather than a stop codon, complete stop codon removal (TAA, TAG, AGA, AGG) and incomplete stop codons (T‐‐, TA‐, A‐‐, AG‐) completed post‐transcriptionally via polyadenylation. Sequences with non ATCG characters or in‐frame internal stop codons are flagged and excluded from downstream analysis. The complete processing logic is shown in Figure 1.

### 3.2 Validation on Real Fish Mitochondrial CDS Sequences

Of the 286 input sequences, 279 (97.6%) were successfully processed and written to the output FASTA file (Figure 2). In these sequences, 169 (59.1%) sequences terminated with a complete stop codon (TAA, TAG, AGA or AGG) and were cleaned by trimming the terminal 3 bp. 110 (38.5%) sequences terminated with an incomplete stop codon (TA‐, TA‐, A‐‐ or AG‐) and were cleaned after the incomplete pattern was recognised. No sequences terminated with TGA (i.e., no RETAINED sequences were found for this dataset, as terminal TGA is very rare in fish mitochondrial PCGs). Seven sequences (22.4%) were marked as SKIPPED due to the presence of in‐frame internal stop codons, which indicates NUMT contamination or annotation errors in source database records. No sequences were assigned NO_STOP status.

**FIGURE 2:**
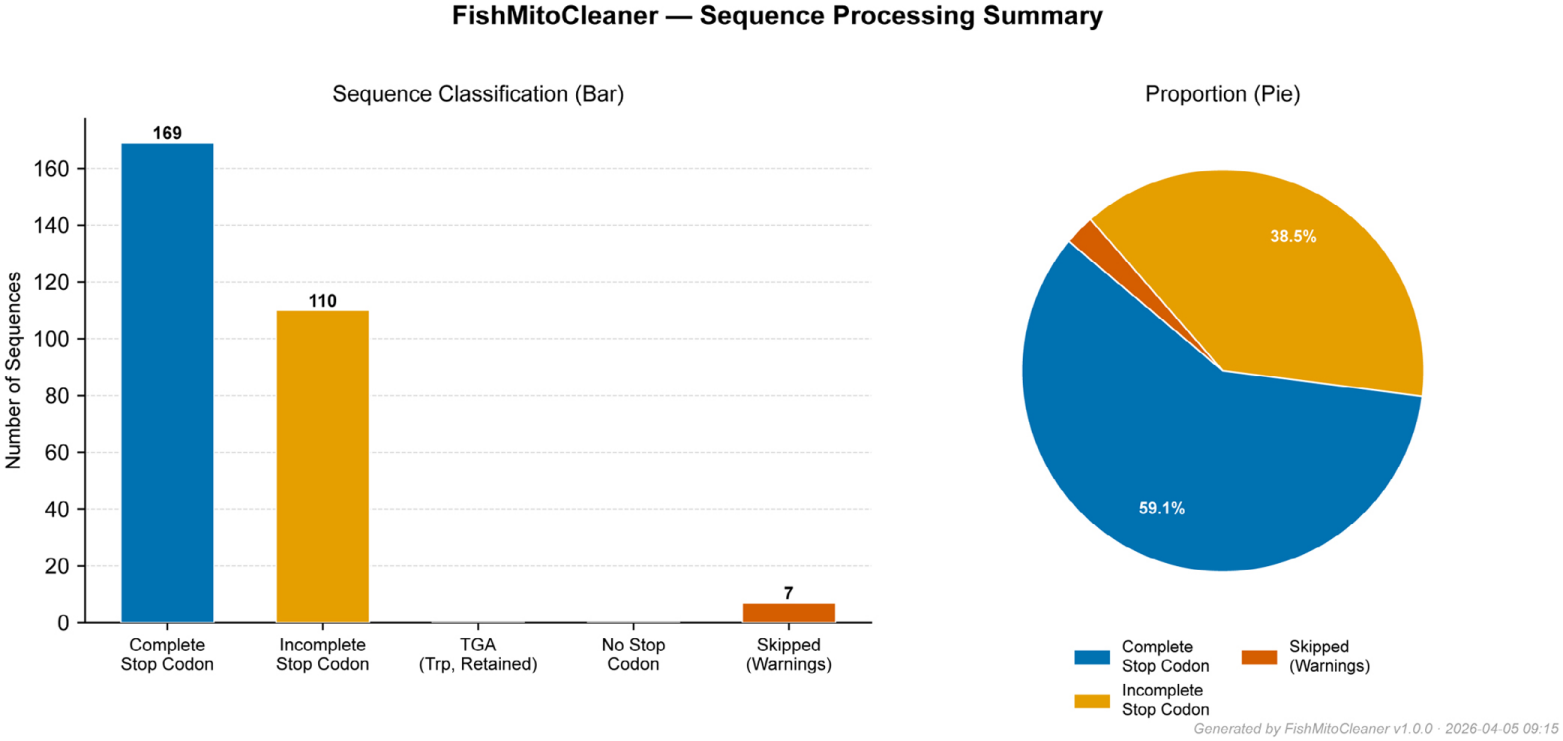
Processing results for the validation dataset (286 CDS sequences from 22 fish species, 7 orders, all 13 mitochondrial PCGs). (Left) Bar chart showing the number of sequences in each processing category. (Right) Pie chart showing the proportional distribution of successfully processed sequences. Of the 286 input sequences, 169 (59.1%) were cleaned after complete stop codon removal, 110 (38.5%) after incomplete stop codon repair and 7 (2.4%) were flagged as SKIPPED due to in‐frame internal stop codons. Figure generated automatically by FishMitoCleaner v1.0.0.

### 3.3 Effect of Cleaning on Codon Usage Analysis

RSCU was chosen as the validation metric because it is sensitive to changes in codon composition caused by stop codon processing. To determine whether trimming performed by FishMitoCleaner had any effect on downstream codon usage, we computed RSCU values for all 60 sense codons of each gene before and after cleaning using the Vertebrate Mitochondrial Code (NCBI Translation Table 2). The mean absolute RSCU difference (mean |RSCU|) between the original and cleaned sequences was calculated for each of the 13 PCGs (Table 2).

**TABLE 2:** Summary of per‐gene sequence counts and mean absolute RSCU differences before and after cleaning with FishMitoCleaner.

Gene	*n* (Original)	*n* (Cleaned)	Mean |ΔRSCU|
*ATP6*	22	21	0.0154
*ATP8*	22	22	0.0000
*COX1*	22	22	0.0000
*COX2*	22	22	0.0000
*COX3*	22	22	0.0000
*CYTB*	22	21	0.0141
*ND1*	22	21	0.0109
*ND2*	22	21	0.0167
*ND3*	22	22	0.0000
*ND4*	22	21	0.0103
*ND4L*	22	21	0.0229
*ND5*	22	21	0.0099
*ND6*	22	22	0.0000

*Note:* RSCU values were calculated using the Vertebrate Mitochondrial Code (NCBI Translation Table 2). Mean |ΔRSCU| = mean absolute difference of RSCU values for all 60 sense codons between original and cleaned sequences. No significant differences were detected for any codon in any gene (Welch's independent samples *t*‐test, all *p* > 0.05, minimum *p* = 0.62).

The mean |RSCU| values for 6 genes (*ATP8*, *COX1*, *COX2*, *COX3*, *ND3*, *ND6*) were exactly 0.0000, meaning cleaning had no effect on the RSCU values of these genes, since all 22 sequences were retained without change in length after cleaning. For 7 genes (*ATP6*, *CYTB*, *ND1*, *ND2*, *ND4*, *ND4L*, *ND5*), one sequence per gene was removed (SKIPPED) and the number cleaned increased to 21, while the mean values of the |RSCU| were very low (0.0099 [*ND5*] to 0.0229 [*ND4L*]). Welch's independent‐samples *t*‐test showed that there were no significant differences in RSCU values between the original and cleaned values for any gene (all *p* > 0.05). These results indicate that FishMitoCleaner does not introduce bias into downstream codon usage analysis.

## 4 Discussion

### 4.1 Principal Findings and Significance

FishMitoCleaner processed 96.6% of input sequences from 286 fish mitochondrial CDS sequences, achieving reliable results over 7 orders and all 13 mitochondrial PCGs. The main strength of FishMitoCleaner is that the tool complies strictly with the vertebrate mitochondrial genetic code (NCBI Translation Table 2), which differs from the standard genetic code in three biologically important ways (Zhu et al. 2023; Brasseur et al. 2023): (i) TGA encodes tryptophan rather than a stop signal; (ii) AGA and AGG function as stop codons; and (iii) incomplete stop codons (T‐‐ and TA‐) are completed post‐transcriptionally by polyadenylation (Osawa et al. 1989). Failure to account for these features when preprocessing sequences (e.g., using standard codon tools) can cause silent but consequential errors in downstream analysis such as codon usage profiling, Ka/Ks estimation and phylogenetic reconstruction. FishMitoCleaner addresses all three issues in a single automated pipeline, which is the main contribution.

### 4.2 Interpretation of Validation Results

The fact that 38.5% of sequences in our dataset contain incomplete stop codons indicates the presence of polyadenylation‐completed termination in vertebrate mitogenomes (Ojala et al. 1981; Donath et al. 2019) and is also consistent with observations in many teleost studies (e.g., 7 of 13 PCGs in the cyprinid *Microphysogobio elongatus* terminate with T or TA (Zhang, Zhu, Li, and Deng 2023), and similar patterns are found in *Antarctic notothenioids* (Papetti et al. 2021) and Mahseer species (Chowdhury et al. 2024)). Our results also reflect genuine biological characteristics of fish mitochondrial CDS, rather than dataset‐specific artefacts. Our result confirms that any preprocessing pipeline for fish mitochondrial CDS data should handle incomplete stop codons as a standard step rather than an exceptional case.

The seven sequences which are flagged as SKIPPED (2.4%) due to in‐frame internal stop codons highlight a practical data quality problem well known elsewhere: public databases such as GenBank contain a non‐negligible proportion of mitochondrial sequences likely to be NUMT inserts or annotation errors (Donath et al. 2019). Our tool provides a simple and transparent way to identify and exclude such sequences, protecting downstream analyses from non‐functional copies.

The RSCU comparison (Table 2) showed that cleaning operations had no significant effect on codon usage, and the largest mean |RSCU| was 0.0229 (*ND4L*), and no significant differences were observed for any gene or codon (all *p* > 0.05). These results strongly support the validity of FishMitoCleaner as a preprocessing step before codon usage analysis, which is one of its main purposes.

### 4.3 Tool Design and Accessibility

The FishMitoCleaner interface was designed to be accessible for researchers in aquatic biology and fisheries science who routinely work with mitogenomes but lack the programming skills to write custom sequence processing scripts. By capturing the full preprocessing logic in one command line call with reasonable defaults and built‐in demonstration mode, the technical barrier of manual inspection or informal scripts is eliminated for many researchers. Automatic generation of a per‐sequence processing report and publication quality statistics reduces the time spent documenting and distributing results, which is often overlooked but increasingly expected by journals with data transparency requirements.

### 4.4 Relationship to Existing Tools

There are several tools for related but distinct tasks in mitochondrial genomics. MitoAnnotator and MITOS annotation complete mitogenome assemblies (Donath et al. 2019; Iwasaki et al. 2013); MitoGeneExtractor extracts target genes from raw sequencing libraries (Brasseur et al. 2023). None of these tools performs the task addressed by FishMitoCleaner: batch quality control and normalisation of already‐extracted, already‐annotated CDS sequences and interacting explicitly with the vertebrate mitochondrial genetic code. This means FishMitoCleaner complements rather than replaces existing annotation and extraction workflows, occupying the step between database download and downstream analysis that is handled inconsistently in the literature.

### 4.5 Extensibility to Other Vertebrates

FishMitoCleaner, validated using fish data, is also applicable to mitochondrial CDS sequences from any vertebrate class because the vertebrate mitochondrial genetic code (NCBI Translation Table 2) is shared among all vertebrate classes (mammals, birds, reptiles and amphibians). As sequencing costs continue to drop and mitogenome databases grow (Veríssimo et al. 2025; Alvarenga et al. 2024), the number of publicly available vertebrate CDS sequences which need preprocessing will grow. FishMitoCleaner provides a ready‐to‐use solution that can be applied directly to these data sets without modification.

### 4.6 Limitations and Future Directions

FishMitoCleaner has three major limitations: FishMitoCleaner implements only the vertebrate mitochondrial code and cannot be applied to other invertebrate sequences. FishMitoCleaner uses pre‐extracted CDS sequences and does not perform genome annotation or gene boundary prediction from raw mitogenomes. NO_STOP sequences are retained with a warning but need to be inspected manually. Additionally, our validation was based on 22 species with RSCU as the primary metric. Future work should include more species, especially those with known mitogenome structural variations, and complementary metrics such as Ka/Ks ratios and amino acid composition analysis. Moreover, naturally occurring insertions or deletions within coding regions may produce sequences whose lengths are not multiples of three. In such cases, FishMitoCleaner will trim the trailing nucleotides to enforce this requirement, which may not be biologically appropriate. Users should inspect such sequences individually. FishMitoCleaner will address these limitations in future versions by supporting additional mitochondrial codes, annotation pipelines and output formats compatible with downstream analysis platforms.

## 5 Conclusion

FishMitoCleaner is a practical, reproducible and easy‐to‐use solution for preprocessing fish mitochondrial protein‐coding CDS sequences with correct handling of the vertebrate mitochondrial genetic code and no bias in downstream codon usage analysis. FishMitoCleaner reduces the technical barrier for aquatic scientists and fisheries scientists, and can be used for mitochondrial CDS data from other vertebrate species.

## Author Contributions

**Shaoyu Yang:** conceptualization (equal), data curation (equal), formal analysis (equal), funding acquisition (equal), methodology (equal), software (equal), validation (equal), writing – original draft (equal), writing – review and editing (equal). **Peng Xu:** data curation (equal), formal analysis (equal), writing – review and editing (equal). **Jingzhen Wang:** funding acquisition (equal), supervision (equal), writing – review and editing (equal). **Xiaohui Cai:** funding acquisition (equal), supervision (equal), writing – review and editing (equal).

## Funding

This work was supported by the Guangxi Natural Science Foundation (no. 2026GXNSFHA00640264), Guangxi Key Research and Development Program (no. GuikeFN2600640277 and GuikeFN2600640574), Chinese White Dolphin Conservation Action Plan (no. 171821301354051004, 171721301354051016) and Guangxi Young Elite Scholar Program.

## Conflicts of Interest

The authors declare no conflicts of interest.

## Supporting information


**Table S1:** List of fish species used in this study.

## Data Availability

The data and source code used in this study can be accessed via the permanent Zenodo link: https://doi.org/10.5281/zenodo.19466490. A secret preview link for reviewers to access the draft deposit is available: https://zenodo.org/records/19466490?token=eyJhbGciOiJIUzUxMiJ9.eyJpZCI6IjYwMTVmZGFiLWY4MWItNDhmZS1iY2Y2LTZlOWI1MGJlMWRlNyIsImRhdGEiOnt9LCJyYW5kb20iOiJiODNiZWY2NWJhMDY5NTJiOGY4Yzk3NDVmNjUxNTgxNyJ9.XrK8Xh2JXPwvpru8xXGIu_IETz6aLzHfrNVx2eMhylodF2_Dpcxpiwls10ChMYT8A0CHN5uVsRjr4u2RBpL5zA. The repository will be published publicly upon acceptance. The mitochondrial CDS sequences used in the validation dataset were downloaded from NCBI GenBank; the corresponding accession numbers are listed in Table S1.
