# Supplementary material for: FishMitoCleaner: Automated Stop Codon Processing and Quality Control of Fish Mitochondrial Protein‐Coding Sequences
Source: Ecol Evol. 2026 Oct 18;16(10):e74557. doi: 10.1002/ece3.74557 (PMC13677282; doi:10.1002/ece3.74557)
Supplement: Supplementary file 1 [file ECE3-16-e74557-s001.docx]

**Table S1. List of fish species used in this study.**

| **No.** | **Species** | **Order** | **GenBank Accession No.** |
| --- | --- | --- | --- |
| 1 | *Danio rerio* | Cypriniformes | NC_002333.2 |
| 2 | *Cyprinus carpio* | Cypriniformes | NC_001606.1 |
| 3 | *Ctenopharyngodon idella* | Cypriniformes | NC_010288.1 |
| 4 | *Cobitis tetralineata* | Cypriniformes | NC_088430.1 |
| 5 | *Schizothorax fedtschenkoi* | Cypriniformes | NC_087781.1 |
| 6 | *Brachymystax lenok* | Salmoniformes | NC_018341.1 |
| 7 | *Oncorhynchus mykiss* | Salmoniformes | NC_001717.1 |
| 8 | *Salmo salar* | Salmoniformes | NC_001960.1 |
| 9 | *Anguilla anguilla* | Anguilliformes | NC_006531.1 |
| 10 | *Anguilla japonica* | Anguilliformes | NC_002707.2 |
| 11 | *Ictalurus punctatus* | Siluriformes | NC_003489.1 |
| 12 | *Silurus asotus* | Siluriformes | NC_015806.1 |
| 13 | *Tachysurus fulvidraco* | Siluriformes | NC_015888.1 |
| 14 | *Epinephelus coioides* | Perciformes | NC_011111.1 |
| 15 | *Acanthopagrus schlegelii* | Perciformes | NC_018553.1 |
| 16 | *Oreochromis niloticus* | Perciformes | NC_013663.1 |
| 17 | *Thunnus albacares* | Perciformes | NC_014061.1 |
| 18 | *Cynoglossus semilaevis* | Pleuronectiformes | NC_012825.1 |
| 19 | *Paralichthys olivaceus* | Pleuronectiformes | NC_002386.1 |
| 20 | *Scophthalmus maximus* | Pleuronectiformes | NC_013183.1 |
| 21 | *Takifugu niphobles* | Tetraodontiformes | NC_011625.1 |
| 22 | *Takifugu rubripes* | Tetraodontiformes | NC_004299.1 |
